# Modulating extracellular matrix stiffness: a strategic approach to boost cancer immunotherapy

**DOI:** 10.1038/s41419-024-06697-4

**Published:** 2024-05-01

**Authors:** Zizhao Mai, Yunfan Lin, Pei Lin, Xinyuan Zhao, Li Cui

**Affiliations:** Stomatological Hospital, School of Stomatology, Southern Medical University, Guangzhou, 510280 Guangdong China

**Keywords:** Cancer immunotherapy, Cancer

## Abstract

The interplay between extracellular matrix (ECM) stiffness and the tumor microenvironment is increasingly recognized as a critical factor in cancer progression and the efficacy of immunotherapy. This review comprehensively discusses the key factors regulating ECM remodeling, including the activation of cancer-associated fibroblasts and the accumulation and crosslinking of ECM proteins. Furthermore, it provides a detailed exploration of how ECM stiffness influences the behaviors of both tumor and immune cells. Significantly, the impact of ECM stiffness on the response to various immunotherapy strategies, such as immune checkpoint blockade, adoptive cell therapy, oncolytic virus therapy, and therapeutic cancer vaccines, is thoroughly examined. The review also addresses the challenges in translating research findings into clinical practice, highlighting the need for more precise biomaterials that accurately mimic the ECM and the development of novel therapeutic strategies. The insights offered aim to guide future research, with the potential to enhance the effectiveness of cancer immunotherapy modalities.

## Facts

1. Extracellular matrix (ECM) stiffness plays a critical role in promoting cancer initiation and progression by regulating the malignant behaviors of cancer cells.
2. Key factors regulating ECM remodeling include the activation of cancer-associated fibroblasts and the accumulation and crosslinking of ECM proteins.
3. ECM stiffness forms a formidable physical barrier around tumors, hindering immune cell infiltration and impeding the precise delivery of immunotherapeutic agents.

## Open questions

1. What is the role of ECM stiffness in altering the tumor microenvironment, and how does it subsequently affect tumor progression and therapeutic outcomes?
2. What are the effects and underlying mechanisms of ECM stiffness on immune cell behaviors within the tumor microenvironment?
3. How can targeting ECM stiffness be optimized to achieve maximal efficacy in various types of cancer immunotherapy?

## Introduction

The process of tumorigenesis depends on two primary factors: genetic and epigenetic alterations within tumor cells, and the dynamic interactions within the tumor microenvironment (TME). The immunosuppressive properties of the TME are crucial in dampening the host’s immune response. The TME includes blood vessels, immune cells, stromal cells, and the extracellular matrix (ECM). Notably, the ECM consists of proteins, mainly divided into fibrous proteins and glycosaminoglycans (GAGs), providing both structural and biochemical support to the resident cells [1, 2]. ECM stiffness, a critical biomechanical property, denotes the resistance of the ECM to deformation when subjected to mechanical stress. It is quantitatively assessed in terms of the elastic modulus. In a physiological context, the rigidity of the ECM is finely balanced, integral to maintaining cellular homeostasis and tissue architecture. However, aberrant alterations in ECM stiffness are implicated in many pathological conditions, particularly in cancer. The ECM dynamically adapts to the progression of cancer, exhibiting shifts in its composition, topological structure, directionality, and notably, its mechanical properties, which directly influence ECM stiffness [3]. These alterations in ECM stiffness subsequently drive changes in cellular mechanobiology, which includes modifications in cytoskeletal tension and the activation of mechanotransduction signaling pathways. Such transformations are pivotal in the evolution of tumors, contributing to their progression towards a more malignant state [4].

Immunotherapy, leveraging the host’s immune system to target malignant cells, has significantly transformed cancer treatment. Compared to traditional therapies such as chemotherapy, this approach exhibits fewer off-target effects and has substantially improved outcomes in various advanced malignancies. However, tumor cells utilize various strategies, including increased ECM stiffness, to impair the efficacy of immunotherapy, leading to inadequate clinical responses in some patients. For instance, elevated ECM stiffness forms a formidable physical barrier around tumors, hindering immune cell infiltration and hampering precise delivery of immunotherapeutic agent. Moreover, increased ECM stiffness can amplify cancer’s immune evasion by altering the expression of immune checkpoint (IC) molecules like PD-L1, PD-1, and CTLA-4, potentially resulting in the failure of IC blockade (ICB) therapy.

Although several reviews have addressed the clinical relevance of the ECM in cancer therapy, the distinctive contributions of our review are noteworthy for a variety of reasons [5–7]. First, it offers a timely and comprehensive synthesis of the latest research exploring the impact of ECM stiffness on tumor and immune cell behaviors. Second, the review extensively discusses the effects of targeting ECM stiffness to enhance the efficacy of a range of immunotherapeutic strategies, including ICB therapy, adoptive cell therapy (ACT), oncolytic virus therapy (OVT), and therapeutic cancer vaccines (TCVs). This thorough analysis presents a multifaceted perspective on the potential of ECM modulation in cancer treatment. Lastly, it underscores the emerging challenges and introduces innovative considerations for targeting ECM stiffness, offering novel insights into how this approach could be optimized to improve the efficacy of immunotherapy.

## The key factors for regulating ECM remodeling

**Fig. 1:**
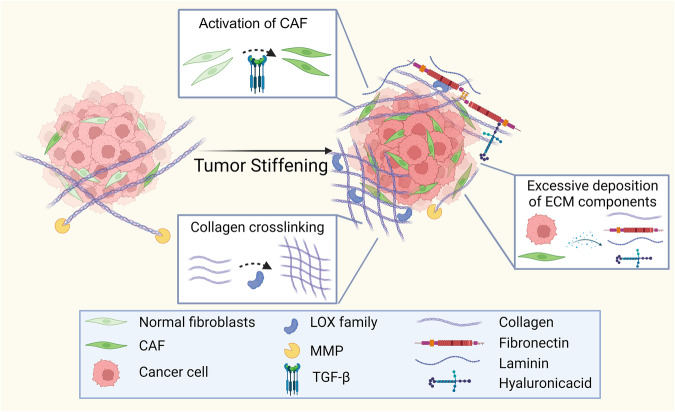
Key factors driving ECM stiffness. The primary contributors to ECM stiffness include the activation of CAFs, excessive deposition of ECM components, and enhanced collagen crosslinking.

The increased stiffness in tumors primarily arises from ECM remodeling processes. Enzymes such as matrix metalloproteinases (MMPs), the lysyl oxidase (LOX) family, and Procollagen-Lysine,2-Oxoglutarate 5-Dioxygenase (PLOD) family play a pivotal role in this remodeling. Key factors that regulate ECM remodeling include the activation of cancer-associated fibroblasts (CAFs) and the accumulation and crosslinking of ECM proteins (Fig. 1) [1].

### Activation of CAFs

Within the TME, fibroblasts frequently undergo a transition into a persistently activated state known as CAFs. This transformation is induced by growth factors secreted by cancer cells, including transforming growth factor-beta (TGF-β), epidermal growth factor, and bone morphogenetic protein. CAFs exhibit a distinct elongated morphology and lack the markers typical of epithelial cells, endothelial cells, and leukocytes. Importantly, they do not carry the genetic mutations characteristic of cancer cells [8]. Central to the synthesis of various ECM components, CAFs also respond to alterations in the ECM’s physical properties by converting mechanical signals into biochemical responses, which subsequently regulate the expression of specific genes and growth factors.

TGF-β plays a crucial role in the transformation of fibroblasts into CAFs, with integrins being essential for TGF-β activation. Notably, the expression of integrin αvβ6 in cancer cells activates TGF-β, which is positively correlated with increased levels of CAF markers such as α-SMA and fibroblast activation protein (FAP) [9]. The knock-out of the integrin α3 subunit can revert CAFs to an inactivated fibroblast state, effectively reducing cancer cell invasion [10]. Beyond TGF-β1, SPIN90 and specific pro-inflammatory cytokines are critical for CAF activation. A decrease in SPIN90 levels leads to increased microtubule acetylation in CAFs, thereby promoting their activation. Furthermore, reduced expression of SPIN90 activates CAFs through the periostin-FAK-ROCK signaling pathway [11]. Additionally, the pro-inflammatory cytokine IL-1α is capable of reprogramming standard fibroblasts into CAFs, which subsequently results in increased matrix stiffness [12].

### Excessive deposition of ECM components

As ECM stiffness increases, there is an accumulation of various ECM components, particularly collagen. In cancer cells, the methylation of the RASSF1A promoter triggers a sequence of events where increased expression of nuclear YAP1 and P4HA2 leads to enhanced collagen deposition in the ECM, thereby contributing to its increased stiffness [13]. HSP47, an endoplasmic reticulum molecular chaperone, facilitates procollagen folding and maturation; its upregulation accelerates collagen secretion and deposition [14]. Beyond collagen, other components such as fibronectin (FN), laminin, elastin, and GAGs significantly modulate ECM stiffness. CAFs produce an FN-rich ECM, with factors like obesity further exacerbating FN accumulation and thereby increasing ECM stiffness [15, 16]. Additionally, ROCK (Rho kinase), acting as a mechanosensor for matrix stiffness, augments tissue stiffness by regulating the synthesis of collagen, FN, and laminin through the β-catenin signaling pathway [3]. An increase in GAG content, induced by YAP activation, leads to increased ECM stiffness, whereas a reduction in GAGs decreases stiffness [17, 18]. Hyaluronic acid (HA), prevalent in the ECM of many fibrosis-associated tumors, profoundly influences their biochemical and mechanical properties. Elevated HA levels are linked with vascular collapse, hypoxia, drug resistance, and a more aggressive tumor phenotype. In metastatic colorectal cancer patients, increased HA and sulfated GAGs (sGAG) following anti-VEGF therapy contribute to enhanced ECM stiffness, creating a physical barrier that hinders drug delivery [19].

Protease families, such as MMPs, adamalysins, and meprins, also play a critical role in ECM remodeling [20]. In cancer, proteases not only facilitate tumor growth and metastasis by altering the ECM structure but also release bioactive ECM fragments to reshape the TME [21]. Specifically, the MMP family, with over 20 zinc-dependent endopeptidases [22], is tightly regulated by tissue inhibitors of metalloproteinases (TIMPs). This regulation is vital in balancing ECM degradation and synthesis [23]. MMPs, particularly MMP-9 and MMP-2, are instrumental in cancer-related processes. For instance, MMP-9-mediated degradation of FN alters αvβ6 integrin dynamics, enhancing β6 integrin expression and promoting cancer cell invasion [24]. Similarly, MMP-2 activity, upregulated by TEM1, promotes tumor invasion and metastasis by enhancing ECM adhesion and degradation [25]. Importantly, growth factors released from ECM cleaved by MMPs contribute to tumor development. In colorectal cancer, neutrophil-derived MMP-9 releases VEGF from the ECM by cleaving heparan sulfates, enhancing endothelial cell sprouting and angiogenesis, underlining MMP-9’s critical role in regulating VEGF bioavailability and its impact on tumor vascularization [26].

A disintegrin and metalloproteinases (ADAMs) and ADAMs with thrombospondin motifs (ADAMTS) are key protease families involved in ECM remodeling; ADAMs modulate cell-ECM interactions and signal transduction through the cleavage of cell surface molecules, while ADAMTS specifically target ECM components such as proteoglycans and collagens, directly contributing to ECM structural changes and functional dynamics. For instance, ADAM8 indirectly contributes to the breakdown and restructuring of the ECM by regulating MMP9 expression, orchestrating the dynamic interplay between these crucial components in ECM remodeling [27, 28]. Additionally, other tissue proteases, such as serine, aspartic, and cysteine proteases, also play significant roles in ECM remodeling [29]. For instance, matriptase plays a key role in cancer progression by altering ECM components such as laminin-332, impacting cell motility and epithelial carcinogenesis [30].

### Collagen crosslinking

In the multifaceted stages of collagen synthesis, crosslinking plays a crucial role, organizing procollagen chains into a cohesive network of collagen triple helices. This process begins with lysyl hydroxylases, encoded by the PLOD family, which hydroxylate lysine residues. Following this, the LOX family facilitates the oxidative deamination of lysine and hydroxylysine, forming reactive aldehydes. These aldehydes spontaneously form crosslinks with adjacent lysine or hydroxylysine residues, consequently enhancing ECM stiffness.

Hydroxylysine aldehyde-derived collagen cross-links (HLCC) predominate in high-stiffness tissues like bone, whereas lysine aldehyde-derived collagen cross-links (LCC) are more common in low-stiffness tissues such as connective tissue. PLOD2 promotes collagen crosslinking and hydroxylates terminal peptidyl lysine residues in collagen, leading to a stromal shift marked by increased HLCC and reduced LCC [31]. The LOX family comprises five copper-dependent amino oxidases: LOX and the LOX-like (LOXL) proteins LOXL-1, LOXL-2, LOXL-3, and LOXL-4 [32]. Tumor-initiating cells secrete LOX to catalyze collagen crosslinking, which initiates a series of events including the upregulation of integrin α7, FAK/Src phosphorylation, and ERK1/2 phosphorylation, ultimately resulting in increased matrix stiffness. Similarly, LOXL2 is pivotal in collagen crosslinking and central to the deposition of insoluble collagen during ECM stiffening.

## The effects of ECM stiffness on tumor cells

**Fig. 2:**
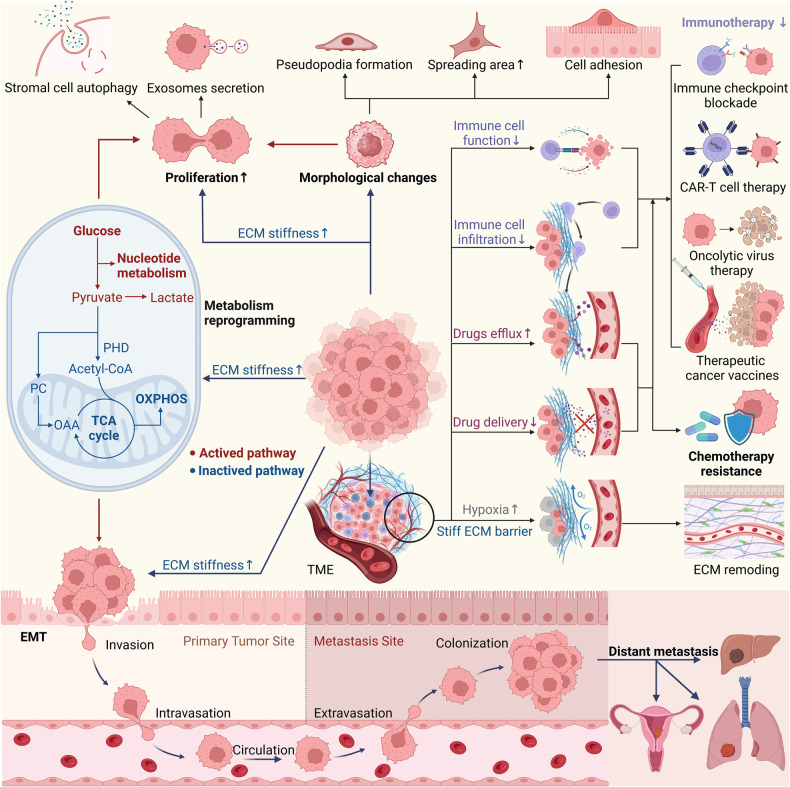
Increased ECM stiffness mediates the aggressive phenotype of cancer cells. Elevated ECM stiffness induces morphological changes in tumor cells, primarily in the form of pseudopodia formation, expanded spreading areas, and enhanced cell adhesion. These changes promote cancer cell proliferation. Additionally, an increase in ECM stiffness drives metabolic reprogramming in cancer cells. Furthermore, EMT facilitated by ECM stiffness contributes to distant metastasis through a sequence of stages. Notably, increased ECM stiffness creates a physical barrier in the TME, which enhances drug efflux, reduces immune cell cytotoxicity, and impedes the infiltration of oxygen, drugs, and immune cells.

The stiffness of the ECM significantly influences the malignant behaviors of cancer cells, including morphological changes, proliferation, metabolic reprogramming, epithelial-mesenchymal transition (EMT), invasion, metastasis, and resistance to chemotherapy (Fig. 2).

### The effects of ECM stiffness on morphology and proliferation of cancer cells

Under conditions of increased ECM stiffness, cancer cells adopt a morphology conducive to adhesion, proliferation, and invasion, thereby facilitating cancer progression. For instance, HCC cells display a compact, rounded shape on softer matrices, whereas on stiffer matrices, they transition to a flattened and extensively spread morphology [33]. Similarly, cells on stiff matrices demonstrate increased spreading, notable actin filament assembly, pronounced pseudopodia formation and enhanced focal adhesion formation [34–37]. Notably, there is a direct correlation between cell morphology and proliferation: expansive cell spreading and pseudopodia extension are crucial for efficient nutrient absorption, leading to increased cell proliferation. Intriguingly, the integrin αV-FAK axis senses ECM stiffness and induces autophagy in stromal cells via AMPKα, supporting the proliferation of adjacent cancer cells [38]. Additionally, increased ECM stiffness activates the FAK-Rho-ERK signaling cascade, leading to MAPK activation. This cascade promotes aberrant proliferation of mammary epithelial cells and heightens susceptibility to breast carcinoma [39].

### The effects of ECM stiffness on metabolic reprogramming of cancer cells

Cancer metabolic reprogramming not only provides energy but also generates essential metabolites necessary for biosynthesis, sustained proliferation, and tumorigenesis. Key metabolic pathways, including aerobic glycolysis, glutaminolysis, macromolecule synthesis, and redox homeostasis, are central to the proliferation and progression of cancer cells [40]. A reciprocal relationship exists between ECM stiffness and cancer cell metabolism. The altered metabolic profiles in cancer cells are associated with ECM remodeling, demonstrating the intricate interplay between tumor mechanical properties and cancer metabolic adaptations. Notably, aspartate derived from CAFs supports cancer cell proliferation, while glutamate from cancer cells maintains the redox balance of CAFs, facilitating ECM modification [41]. Additionally, increased ECM stiffness enhances aerobic glycolysis in cancer cells via YAP activation, contributing to cancer cell migration [42, 43].

### The effects of ECM stiffness on EMT of cancer cells

EMT is a cellular process where polarized epithelial cells, in contact with collagen IV at the basal surface, transform to acquire a mesenchymal phenotype. This transformation is characterized by increased migratory and invasive capabilities, enhanced resistance to apoptosis, and elevated production of ECM components [44]. Signals originating from ECM stiffness initiate EMT, driving the transformation of cell morphology towards a mesenchymal phenotype and enhancing their invasive and migratory behaviors [45]. Specifically, ECM stiffness modulates cancer cell migration and invasion by promoting the expression of N-cadherin, while concurrently reducing the levels of E-cadherin [46–48]. Furthermore, enzymes responsible for ECM remodeling, which often exhibit increased expression during EMT, are prevalent in aggressive cancer phenotypes [49].

### The effects of ECM stiffness on invasion and metastasis of cancer cells

The ECM plays an active role in cancer cell invasion and metastasis processes. Cancer cells, through their interaction with the ECM, are better equipped to invade the surrounding stroma. MMPs, particularly MMP-2 and MMP-9, are key in governing cancer invasion and metastasis by degrading the ECM and promoting EMT. Elevated ECM stiffness activates the FAK/RhoA/ROCK and PI3K/AKT signaling pathways via integrins, thereby increasing the expression of MMP2 and MMP9, and enhancing the invasiveness of cancer cells [50]. Importantly, the role of MMP-9 in response to ECM stiffness is notably context-dependent. In cancer, increased ECM stiffness often initiates EMT, closely linked with MMP-9 upregulation, thereby facilitating tumor invasion [51]. In contrast, the same increase in ECM stiffness can lead to reduced MMP-9 activity in liver fibrosis, serving as a regulatory mechanism to limit ECM degradation and maintain fibrotic tissue structure [52]. Interestingly, in hepatocellular carcinoma (HCC) cells, higher matrix stiffness can enhance EMT and increase MMP-9 expression [46], suggesting a divergent role of MMP-9 from liver fibrosis to HCC progression. This highlights the importance of cell-specific responses, tissue-specific environments, and differential signaling pathways in determining MMP-9’s function across various stages and conditions.

Cancer metastasis, involving the spread of cancer cells from the primary tumor to distant tissues and organs, primarily encompasses five stages: invasion, intravasation, circulation, extravasation, and colonization. Colorectal liver metastasis is a prominent example of distant metastasis influenced by ECM stiffening. The presence of CAFs in the TME is more pronounced in patients with colorectal cancer liver metastasis, correlating with the role of the fibrosis marker LTBP2 in collagen synthesis and ECM restructuring [53, 54]. Similarly, TCF21 increases perivascular ECM stiffness in peritumoral cells, creating a metastatic microenvironment conducive to stimulating colorectal cancer liver metastasis [53, 55].

### The effects of ECM stiffness on drug resistance of cancer cells

Recurring tumors post-treatment often exhibit drug resistance, characterized by significant changes in tumor cell differentiation, immune infiltration, and ECM composition. The stiffness of the ECM plays a crucial role in determining the responsiveness of cancer cells to chemotherapy. For instance, elevated ECM stiffness enhances the activity of the multidrug resistance protein 1 on cell membranes, increasing its ability to expel drugs from cancer cells and thereby conferring chemotherapy resistance [56]. Furthermore, increased tumor stiffness obstructs the efficient delivery of therapeutic drugs within the tumor, diminishing the efficacy of such treatments [57].

## The crucial role of biomechanical forces in regulating immune cell function

**Fig. 3:**
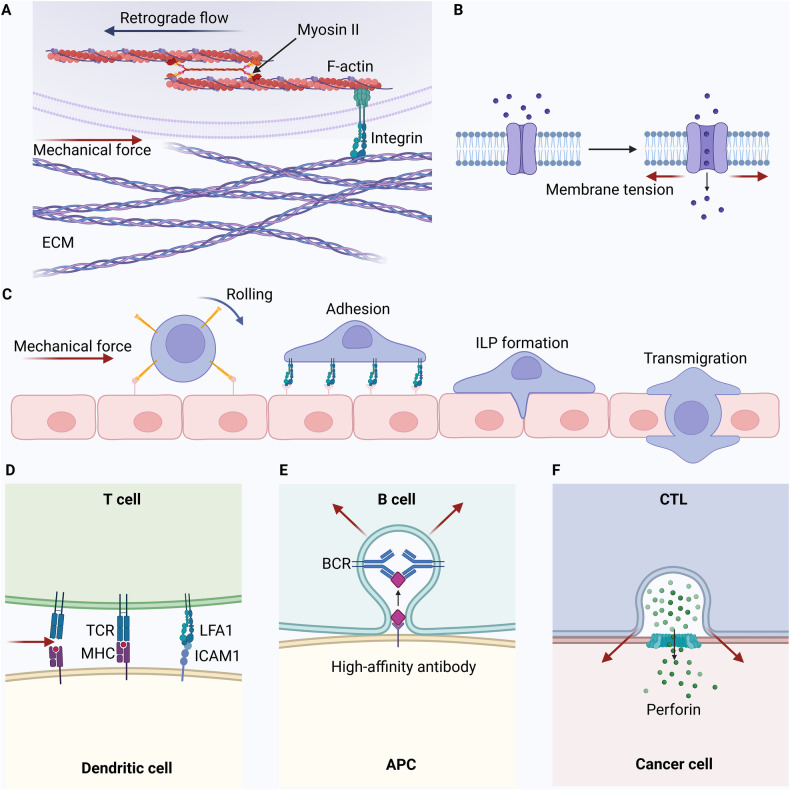
Mechanical forces influence immune cell movement and functionality. **A** Force changes detected by immune cell integrins drive movement through retrograde F-actin flow, connected by myosin II. **B** Membrane tension opens ion channels on immune cells, converting extracellular mechanical cues into intracellular biochemical signals via ion influx. **C** Under force, immune cells connect to epithelial cells via selectins and roll on their surfaces. At specific sites, immune cell integrins bind to receptors on epithelial cells. Post-adhesion, immune cells develop invadopodia-like protrusions (ILPs), aiding in transmigration. **D** Antigen recognition necessitates direct contact between T cells and APCs. Mechanical forces influencing receptor-ligand bond formation affect the formation of the immunological synapse. LFA1, the primary integrin on T and B cells, binds tightly to adhesion molecules ICAM1 and ICAM2 on APCs, governing mechanosensitive antigen recognition. **E** B cells selectively internalize high-affinity antigens via myosin IIa-driven contraction, pulling and invaginating the presenting membranes. **F** CTLs tighten synapses on target cell membranes by applying force, promoting perforin’s pore-forming activity, and enhancing cytotoxicity.

The innate immune arm comprises cells such as macrophages, neutrophils, dendritic cells (DCs), mast cells, basophils, eosinophils, natural killer (NK) cells, and innate lymphoid cells. On the other hand, adaptive immunity primarily involves antigen-specific T cells, with B cells differentiating into plasma cells to produce specific antibodies. Notably, biomechanical forces between immune cells are crucial for maintaining their functions (Fig. 3). T cells are regulated by biomechanical forces that affect their migration, recognition, activation, and effector functions [58]. Furthermore, antigen recognition requires direct physical interactions between T cells and antigen-presenting cells (APCs). During this process, biomechanical forces can modify receptor-ligand bond interactions, affecting the formation of the immunological synapse [59]. LFA1, a key integrin on T and B cells, interacts with adhesion molecules ICAM1 and ICAM2 on APCs, modulating the mechanosensitive antigen recognition process. In addition, B cells selectively internalize high-affinity antigens via contraction mediated by dynamic myosin IIa, influencing the presenting membranes [60]. Similarly, the synapse on CTLs increases the membrane tension of the target cell, enhancing the activity of perforin released by CTLs and amplifying their cytotoxicity [61].

Cells interact with the ECM, and biomechanical forces generated by ECM directly or indirectly influence immune cell fate and function. Therefore, elucidating the relationship between biomechanical force and ECM stiffness is essential in determining cellular responses. In this dynamic, the biomechanical force is primarily a consequence of the ECM’s stiffness, exerted upon the cells [62]. A stiffer ECM provides greater mechanical resistance, which cells sense and respond to. This interaction is critical for mechanotransduction, where cells interpret these mechanical cues from the ECM and translate them into biochemical signals [63]. This process significantly influences cellular behaviors. For instance, the biomechanical force derived from stiff lymph nodes, sensed by the mechanosensor YAP, suppresses T cell proliferation by inhibiting NFAT1’s nuclear translocation [64].

## The effects of ECM stiffness on immune cells

The stiffness of ECM profoundly influences the activation, polarization, migration, infiltration, antigen presentation, and cytotoxicity of immune cells, ultimately affecting the efficacy of cancer immunotherapy. Stiffer tumors often display decreased immune cell density at their core compared to softer ones. The migration of immune cells within the ECM is governed by the interplay and spatial arrangement of its constituents, with collagen fibers playing a critical role [65]. Elevated ECM stiffness and dense collagen networks can impede immune cell infiltration and motility, thereby diminishing the cytotoxic interactions between immune and cancer cells [66].

### The effects of CAFs on immune cells

It is well documented that CAFs play a pivotal role in modulating ECM stiffness and impacting immune cell function [67]. They contribute to immune evasion by creating a physical barrier that impedes immune cell infiltration and by secreting a range of cytokines and chemokines that can alter immune cell recruitment, activation, and function [6]. For instance, CAFs in esophageal cancer negatively correlate with CD8^+^ TILs and positively with FoxP3^+^ TILs, indicating a role in tumor immunosuppression. Elevated IL6 secretion in CAF-cancer cell cocultures leads to altered TIL profiles, with IL6 blockade reversing these effects [68]. Similarly, CAFs directly suppress cytotoxic T cells, using mechanisms like antigen processing, cross-presentation, and PD-L2 and FASL-mediated killing. This suppression is evident in human tumors and can be counteracted in vitro and in vivo by neutralizing PD-L2 or FASL [69]. Notably, CD70 expression on a specific subset of CAFs in invasive CRC serves as an independent adverse prognostic marker. These CD70-positive CAFs facilitate tumor migration and increase Treg frequency, contributing to immune escape in CRC and underscoring the potential of CD70-targeting antibodies in therapy [70]. In oral squamous cell carcinoma, the CD68+ CAFs vary in distribution within the tumor and are involved in immune modulation, notably by enhancing Treg recruitment through chemokines CCL17 and CCL22 [71].

In addition to T cells, CAFs also exert significant influence on other immune cells, such as neutrophils, macrophages, and NK cells, impacting various aspects of the immune response within the TME. CAFs influence neutrophil chemotaxis, survival, and activation in HCC mediated by an IL6-STAT3-PDL1 signaling pathway [72]. This interaction impairs T-cell function, underscoring a novel mechanism of CAFs in tumor microenvironment modulation and identifying a potential therapeutic target in HCC. In breast cancer, CAFs drive monocyte recruitment and polarization towards immunosuppressive M2-like macrophages, enhancing cancer progression and shaping an immunosuppressive microenvironment [73]. Interestingly, in colorectal cancer, CAFs promote monocyte adhesion and M2 macrophage polarization through IL-8 secretion and VCAM-1 upregulation, working synergistically with TAMs to suppress NK cell function [74]. In addition, CAFs in HCC induce NK cell dysfunction, characterized by reduced cytotoxicity and impaired cytokine production, through PGE2 and IDO [75]. Importantly, in esophageal squamous cell carcinoma, CAFs facilitate the differentiation of monocytes into M-MDSCs, enhancing drug resistance via IL-6 and miR-21-mediated STAT3 activation [76]. This interaction between CAFs and M-MDSCs correlates with poorer patient survival, highlighting a potential target in STAT3 signaling for overcoming chemotherapy resistance.

### The effects of ECM stiffness on T cell functionalities

T cells recognize peptide antigens derived from intracellular protein degradation and are classified into two main subsets based on surface glycoproteins: CD4^+^ and CD8^+^ T cells. CD8^+^ T cells, also known as CTLs, directly target and eliminate virus-infected and cancerous cells. CD4^+^ T cells, often referred to as “helper T cells,” assist in amplifying the immune response by activating memory B cells and CD8^+^ T cells. The adaptive immune response’s efficacy in preventing tumor formation relies on the ability of CTLs to identify foreign or mutated antigens on transformed cells and subsequently induce cell death via T-cell-mediated cytotoxicity.

Increased ECM stiffness can impair T cell migration and infiltration by forming barriers through ECM remodeling. For instance, loosely arranged collagen fibers within the ECM create a conducive environment for immune cell migration, facilitating efficient movement of T and B lymphocytes [77]. In addition, attributes of collagen fibers, such as orientation and density, influence the migration patterns of resident CD8^+^ T cells within tumors [78]. Elevated ECM stiffness leads to increased tenascin-C expression, which acts as a physical barrier, hindering T-cell movement and infiltration [79–81]. Moreover, the fibrotic ECM directly impacts T cells, resulting in limited infiltration in solid tumors and a predominantly immunosuppressive TME [82, 83]. Interestingly, βig-h3, predominantly produced in the ECM by CAFs, can be targeted to reduce tumor growth by enhancing the numbers of activated CTLs [84]. Notably, SOD3, a crucial modulator of tumor laminin, facilitates the selective infiltration of T lymphocytes into tumors, transforming inactive tumors into immunologically active ones [85].

In addition to migration and infiltration, ECM stiffness critically regulates T cell including proliferation, differentiation, and cytotoxic function. For example, a less stiff matrix enhances the proliferation of both CD4^+^ and CD8^+^ T cells and increases IL-2 secretion [86, 87]. In addition, signaled through the mechanosensor Piezo1, ECM stiffness influences the differentiation balance between Th1 cells and regulatory T cells (Tregs) [88]. Moreover, for an effective immune response, T cells need to increase nutrient uptake. However, T cells can be deprived of essential nutrients due to ECM stiffness resulting from vascular calcification and metabolic shifts, thereby impeding effector T cell differentiation and function [89]. Importantly, High collagen concentration within tumors correlates with fewer infiltrating CD8^+^ T cells and a higher CD4^+^/CD8^+^ ratio, along with reduced expression of cytotoxic activity markers [86, 90]. Similarly, CTL-based therapies are less effective in collagen-rich environments as ECM density and porosity significantly impact CTL performance [91]. Interestingly, microtubule inhibitors like colchicine can improve the cytotoxic efficiency of CTLs in collagen-rich environments [91]. Notably, the use of TGF-β blockade antibodies in combination with anti-PD-L1 antibodies reduces TGF-β signaling in stromal cells, thereby enhancing T cell infiltration and triggering potent anti-tumor immune responses [92].

### The effects of ECM stiffness on tumor-associated macrophages

Macrophages, originating from monocytes in the bone marrow, enter the bloodstream as monocytic precursors and differentiate into fully mature macrophages upon infiltrating tissues and organs. These cells can be categorized into two distinct phenotypes: “classically activated macrophages” or M1, exhibiting a pro-inflammatory phenotype, and “alternatively activated macrophages” or M2, known for their anti-inflammatory characteristics. M1-like macrophages release molecules such as reactive oxygen intermediates, reactive nitrogen intermediates, and TNF-α to suppress tumor growth. In contrast, M2-like macrophages facilitate tumor progression and metastasis through the secretion of matrix-degrading enzymes, vascular growth factors, immunosuppressive cytokines, and chemotactic factors.

ECM stiffness significantly influences the morphology, cytoskeletal arrangement, functionality, and metabolism of macrophages. On high-stiffness substrates, macrophages adopt an elongated, spindle-like shape, with an extended spreading area and increased formation of filamentous pseudopodia. Elevated substrate stiffness also enhances the expression of CSF-1 in cancer cells, driving macrophages towards an M2-like phenotype. Conversely, pliable substrates induce reactive oxygen species (ROS) production in macrophages, leading to polarization to the M1 phenotype via the ROS-mediated NF-κB pathway, and resulting in increased secretion of cytokines IL-1β and TNF-α [93, 94]. Additionally, interactions between βig-h3 and type I collagen form denser fibers. Macrophages cultured on these fibers undergo morphological changes, exhibiting an enhanced M2 phenotype and amplified immunosuppressive capabilities [95].

### The effects of ECM stiffness on diverse immune cell function

**Fig. 4:**
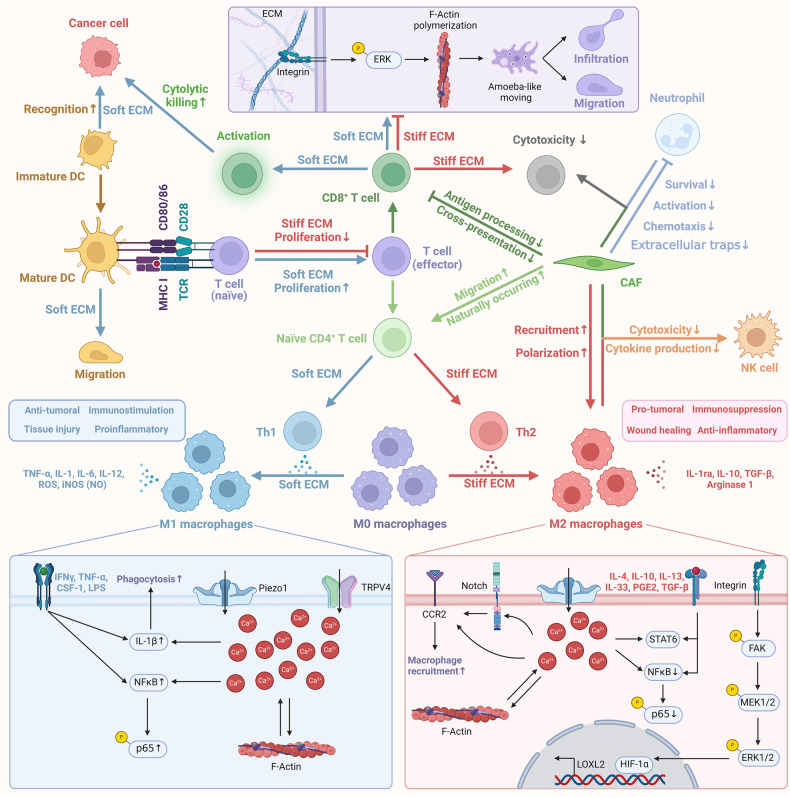
Overview of cellular mechanisms in immune cells influenced by varying ECM stiffness. In soft ECM environments, there is enhanced T cell proliferation and guidance of CD8^+^ T cell activation, migration, and infiltration. Conversely, a stiff ECM hinders T cell proliferation and reduces CD8^+^ T cell mobility and cytotoxicity. Low ECM stiffness steers naive CD4^+^ T cells toward Th1 polarization and promotes M1 macrophage polarization from M0, aiding in anticancer immune effects. High ECM stiffness directs immature CD4^+^ T cells to Th2 polarization and M2 macrophage polarization from M0, fostering pro-cancer TME effects.

The implications of ECM stiffness also extend to various immune cell types. For example, the protein STEAP3, whose activity is influenced by matrix stiffness, facilitates neutrophil infiltration [96]. Upon activation, neutrophils can release chromatin to form neutrophil extracellular traps as part of their immune defense strategy. However, increased matrix stiffness may impair this response [97]. Additionally, SOX9, by increasing collagen deposition and thereby intensifying ECM stiffness, leads to reduced infiltration of DCs within tumors, as well as decreased infiltration of NK and CTL cells [98]. Notably, matrix stiffness influences immature DCs by regulating the expression of β2 integrin and C-type lectin, which affect antigen uptake and podosome formation. It also modulates the expression of CD83 and CCR7 in mature DCs, directing chemokine-driven migration (Fig. 4) [99].

## The effects of ECM stiffness on tumor endothelial cells (TECs) and pericytes

Tumor progression is closely linked with angiogenesis, characterized by the presence of TECs lining the blood vessels, pericytes enveloping the microvasculature, and smooth muscle cells within arterioles and venules. ECM stiffness plays a crucial role in capillary morphogenesis, network development, and barrier integrity, directly affecting the nature and extent of angiogenesis. This active restructuring of the ECM fosters a perivascular niche favorable for cancer metastasis.

TECs, known for their enhanced proliferative, immune-regulatory, and angiogenic capabilities compared to standard endothelial cells, exhibit energy due to their unresponsiveness to pro-inflammatory stimuli. They hinder the adhesion and migration of immune cells, presenting challenges for immune-based cancer therapies. Persistent abnormal physical signals within the TME disrupt the balance between cellular and ECM forces, influencing the gene expression and behavior of TECs. Additionally, tumors stimulate TECs through the secretion of VEGF, which is further enhanced by ECM stiffening. This increased VEGF secretion enhances TEC proliferation and migration. The YAP activity in TECs, influenced by VEGF and mediated by ECM stiffness, promotes endothelial cell proliferation, migration, and sprouting, driving neovascularization [100]. Notably, TECs are highly responsive to changes in ECM stiffness; enhanced traction forces lead to increased cellular contractility, affecting aberrant angiogenesis [101, 102].

Pericytes, integral components of the vascular wall, are embedded within microvascular collagen IV. Dysfunctional interactions between pericytes and TECs can lead to the formation of disorganized, permeable, and ineffective vascular structures within tumors. During ECM fibrosis, increased matrix stiffness may trigger the conversion of pericytes into fibroblasts. This conversion enhances cancer cell mobility and invasiveness through YAP activation [103, 104]. Interestingly, tumor pericytes contribute to perivascular ECM stiffness. They rearrange collagen and degrade collagen IV, influencing ECM remodeling. This remodeling establishes a perivascular environment that is conducive to cancer metastasis [53].

## Targeting ECM stiffness for improving the efficacy of immunotherapy

The clinical achievements of immunotherapy have been both promising and limited. While significant improvements following immunotherapeutic treatments have been observed in a proportion of patients with various cancers, the majority have seen minimal or no benefit from these interventions. The varied responses to immunotherapies are influenced by factors such as tumor heterogeneity, the nature and phase of cancer, the patient’s treatment history, tumor-induced immune suppression mechanisms, and the need to identify specific oncogenic biomarkers and pathways before treatment. Challenges like immune-related side effects, off-target consequences, and immune evasion also persist with immunotherapies.

Single-agent immunotherapy approaches face the challenge of multiple immune resistance mechanisms. This highlights the potential advantage of combination therapies in preventing the emergence of immune-resistant strains. Increased tumor stiffness can impede the effective delivery of immunotherapeutic agents to tumor sites. Recent studies suggest that targeting the tumor ECM with immunotherapies could be particularly effective against treatment-resistant tumors with abundant stroma.

### Targeting ECM stiffness for improving the efficacy of ICB therapy

ICB therapy targets T cell regulatory pathways to boost anti-tumor responses. Normally, immune checkpoints on immune cells prevent excessive activation, protecting healthy tissue. In tumors, however, increased checkpoint expression impairs T-cell activity, weakening the immune response against cancer [7]. Notably, the efficacy of ICB therapy is challenged by the dense ECM in tumors, which can hinder immune cell infiltration and limit therapeutic impact.

ECM stiffness has been shown to critically regulate the expression of IC molecules. For instance, an increase in collagen fiber density, leading to enhanced matrix stiffness, may stimulate PD-L1 expression, thereby promoting immune evasion in cancers [105, 106]. Additionally, a deficiency in SNF5 is associated with an increased presence of tumor-infiltrating CD8^+^ T cells and a reduction in PD-L1-positive cells in vivo. Conversely, increased ECM stiffness can promote cancer cell immune evasion by promoting the activation of the STAT-3 pathway, a consequence of elevated SNF5 expression [107]. Similarly, increased matrix stiffness induces the formation of actin stress fibers, resulting in increased PD-L1 expression in cancer cells. This upregulation can be mitigated using actin polymerization inhibitors [108]. Notably, increased stiffness in the ECM can lead to hypoxia. Under such conditions, HIF-1α, a central transcriptional regulator in the hypoxic response, upregulates PD-L1. Combining HIF-1α inhibitors with anti-PD-L1 antibodies has demonstrated a stronger inhibitory effect on tumor growth [109].

PD-1 is widely expressed on a variety of immune cells, and ECM stiffness is closely associated with PD-1-based immunotherapy. For instance, ECM stiffness can act as an indicator of patient outcomes following treatment with PD-1 inhibitors in combination with Lenvatinib [110]. Additionally, reducing ECM stiffness by targeting collagen crosslinking can enhance T cell migration, thereby improving the efficacy of anti-PD-1 therapies [79]. Moreover, neutrophil elastase, known for its ability to degrade the tumor ECM, facilitates CTL infiltration into tumors and enhances the efficacy of PD-1-based immunotherapy [111]. Interestingly, targeting LOX family members with specific inhibitors can also improve T cell migration, boosting the effectiveness of anti-PD-1 therapies [79]. Similarly, exposing macrophages to LOXL4 can induce an immunosuppressive phenotype and upregulate PD-L1 expression. The synergistic use of ICB and LOXL4 inhibitors presents a promising approach to enhance immunotherapeutic outcomes. Notably, a semiconducting polymer-based agent that reduces ECM stiffness by inhibiting LOX enzyme activity can be combined with immunogenic phototherapy and ICB immunotherapy. This combination augments cytotoxic T cell penetration into tumors, inhibiting tumor proliferation and spread [112]. Furthermore, the simultaneous inhibition of MEK and STAT3 can reprogram CAFs and the immune microenvironment, offering a potential approach to overcome tumor immune resistance to ICIs [113].

**Fig. 5:**
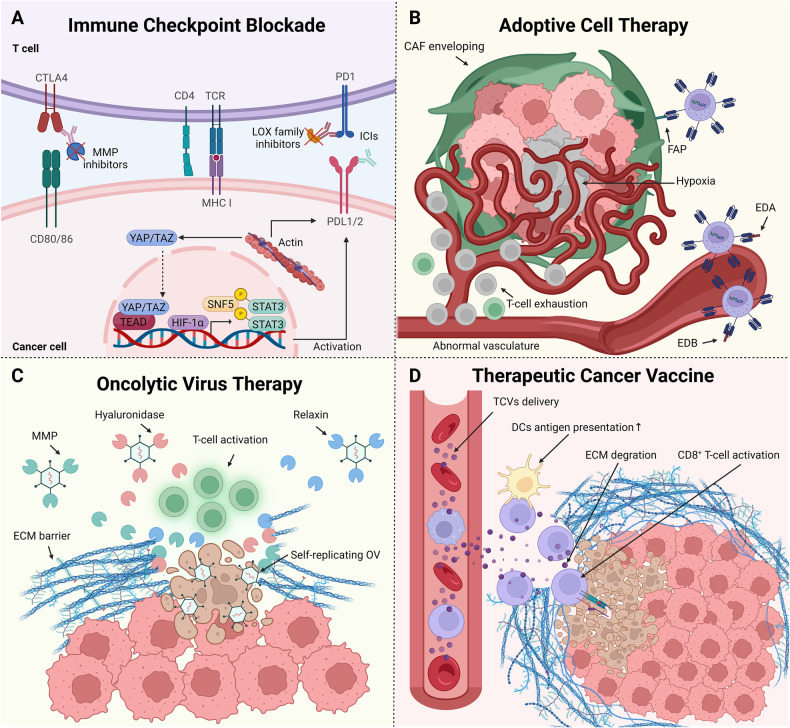
Targeting ECM stiffness for improving the efficacy of immunotherapy. **A** Mechanisms and strategies to counter high ECM stiffness in ICB. Elevated ECM stiffness modulates the expression of ICs through mechanotransduction signaling pathways. ICIs delivery is enhanced when combined with ECM remodeling agents like MMP and LOX inhibitors, enhancing ICB therapeutic efficacy. **B** Physical factors impeding CAR-T cell delivery and corresponding strategies. In tumors with high ECM stiffness, CAF encapsulation and abnormal vasculature obstruct CAR-T cell infiltration and lead to T-cell exhaustion. Targeting specific proteins, such as EDA, EDB, or CAF marker FAP, facilitates CAR-T cell penetration, effectively curtailing tumor growth. **C** Physical barriers to OV delivery and strategies to overcome them. The stiff ECM barrier hinders OVs from reaching tumor cells. Pairing OVs with ECM-degrading proteins, such as MMPs, hyaluronidase, or relaxin, breaks down this barrier, amplifying the OV’s cytotoxic effects by T-cell activation and self-replicating. **D** Therapeutic strategies for overcoming high ECM stiffness impeding TCV delivery. The stiff ECM barrier hampers TCV effects. TCVs combined with ECM-degrading agents promote antigen presentation of DCs and stimulates the activation of CD8^+^ T cell, thereby intensifying their cytotoxicity against cancer cells.

CTLA-4 functions by binding to CD80/86 ligands on CD28, subsequently interacting with the TCR. This interaction leads to the cessation of T cell activation. Initial concentrations of certain collagen proteins in the ECM have been linked to response rates and survival outcomes in patients with metastatic melanoma receiving anti-CTLA-4 therapy [114]. Moreover, MMP and granzyme B-mediated degradation of type III and IV collagens may enhance prognoses [115]. Similarly, small molecule inhibitors targeting MMP2/9 can increase antitumor immunity, reduce tumor burden, and extend patient survival. These inhibitors also effectively reduce PD-L1 expression in cancer cells, thereby enhancing the effectiveness of PD-1 or CTLA-4 therapies (Fig. 5A) [116].

### Targeting ECM stiffness for improving the efficacy of ACT

ACT is a personalized cancer treatment involving the infusion of autologous or allogeneic T cells [117]. Clinical trials have demonstrated significant, sometimes long-lasting, tumor regressions using expanded tumor-infiltrating lymphocytes [118]. The process includes isolating T cells from the patient, engineering them to express chimeric antigen receptors (CARs) or TCRs, cultivating them, and reintroducing them to attack cancer cells [119]. Despite its successes, ACT faces challenges such as antigen escape, effects on normal tissues, limited T cell infiltration into tumors, the suppressive TME, and side effects from modified T cells [120].

The dense structure of the ECM acts as a barrier to CAR-T cell infiltration, due in part to mechanical activation of the TGF-β pathway and abnormal vascular networks [82]. Consequently, CAR-T cells often fail to reach their intended tumor targets, undergo metabolic alterations, and shift towards an immunosuppressive environment, resulting in T cell exhaustion [83]. An innovative approach targeting laminin within the ECM has been shown to enhance immune cell infiltration by degrading the ECM, improving tumor blood flow, and boosting the delivery efficacy of therapeutic agents [121]. Importantly, applications of FN-targeted T cell therapies have shown potential, demonstrating both tolerance and efficacy in advanced cancer cases [122]. Drugs like nattokinase show promise; their administration not only degrades FN but also combats fibrosis enhanced by CAFs, thereby reducing tumor stiffness, enhancing CAR-T cell infiltration, and bolstering the overall efficacy of CAR-T therapy for solid tumors [123]. Specific isoforms of FN, arising from selective mRNA splicing and the addition of structural domains, can influence the outcomes of CAR-T therapy. The extra domain A (EDA) and extra domain B (EDB) are primary FN domains utilized in CAR-T treatments. CAR-T cells targeting EDA have demonstrated anti-angiogenic properties and notable reductions in genes associated with pathways critical to tumor progression, such as EMT, collagen synthesis, IL-6/STAT5, and KRAS pathways [124]. Notably, gold nanoparticles equipped with EDB can induce CTL activation, offering a strategic advantage in cancer immunotherapy [125]. Similarly, CAR-T cell strategies focusing on EDB have shown efficacy in slowing tumor growth through enhanced immune infiltration and promoting tumor cell death [126].

Targeting CAF markers such as FAP with chimeric CAR-T cells has shown promise in limiting tumor growth and enhancing host immunity, while presenting minimal side effects [127]. The introduction of FAP-targeted CAR-T cells has been observed to reduce tumor blood supply and halt tumor growth [128]. In addition, transferring CAR-T cells that target FAP can substantially diminish FAP-expressing stromal cells, leading to inhibition of tumor growth [129]. Furthermore, combining anti-FAP CAR-T cell approaches with PD-1 inhibitors could offer a more effective strategy against tumor progression (Fig. 5B) [130].

### Targeting ECM stiffness for improving the efficacy of OVT

Oncolytic viruses (OVs) target and disintegrate cancer cells through oncolysis, simultaneously activating the host’s immune system for an antitumor response [131]. Importantly, OVs possess a natural preference for cancer cells, or can be genetically modified to target specific tumor markers. Once inside the tumor, OVs attach to cell receptors, using the host cell’s machinery to replicate. The resulting breakdown of infected cells releases new viral particles, extending their anti-tumor action [132]. The clinical application of OVT is limited by the rapid development of neutralizing antibodies and potential risks for immunocompromised patients due to intense immune reactions [133, 134]. Notably, the ECM also poses a barrier to deep tumor tissue penetration of OVs [6].

Current combined OVT approaches with ECM-degrading agents include hyaluronidase, core protein polysaccharides, and relaxin. VCN-01, a specially engineered oncolytic adenovirus, replicates and produces hyaluronidase in cancer cells through the disrupted RB1 pathway. The hyaluronidase released by VCN-01 degrades the tumor stroma, reducing tumor stiffness and facilitating the delivery of various therapeutic agents, including chemotherapy and therapeutic antibodies [135]. Similarly, the oncolytic adenovirus ICOVIR17 promotes hyaluronan degradation in the ECM and increases PD-L1 expression in cancer cells and macrophages, enhancing the infiltration of CD8^+^ T cells and macrophages into the tumor [136]. Core protein polysaccharides, known for degrading ECM components by reducing collagen fiber diameter and modulating TGF-β, also enhance MMP-1 activity. Oncolytic adenoviruses expressing core protein polysaccharides can promote viral spread and elevate their cytotoxic effects against cancer cells [137]. Relaxin, an ECM-degrading protein, improves viral transduction and propagation efficiency, broadens virus distribution and penetration, inhibits tumor growth and metastasis, and improves survival rates post-treatment (Fig. 5C) [138].

### Targeting ECM stiffness for improving the efficacy of TCVs

TCVs, which utilize tumor-associated antigens and tumor-specific antigens, stimulate the immune response against cancer, potentially extending remission and improving survival rates. Currently, TCVs are mainly employed alongside traditional therapies for treating small, residual post-surgery tumors, with their broader application constrained by the evolving nature of cancer cells and the immunosuppressive TME [139]. Furthermore, the ECM presents an additional obstacle, acting as a physical barrier to vaccine delivery. Modulating the ECM could improve vaccine infiltration and reshape the TME, potentially enhancing the effectiveness of TCV therapies.

Vaccines targeting ECM stiffness focus on degrading the ECM to boost T lymphocyte activity and cytotoxicity. For instance, TCVs containing the Spam1 gene express hyaluronidase, degrading the ECM, enhancing blood flow, reducing tumor hypoxia, and modulating the immune environment, thereby elevating antitumor efficiency and reducing tumor recurrence risk [140]. In addition, TCVs targeting the EDA domain of FN have shown potential in curbing metastatic tumor progression [141], while those targeting the EDB of FN effectively diminish the bulk of solid tumors [142–144]. Moreover, an orally administered DNA vaccine targeting FAP-depleted CAFs decreases type I collagen content, restricting the growth and metastasis of primary tumor cells promoting multidrug resistance in mice [145]. Combined with chemotherapy, this vaccine amplifies anti-tumor effects by increasing chemotherapy drug uptake and accumulation [145, 146]. Likewise, clinical research on a FAP-targeted cancer vaccine has shown promising safety profiles and tumor engagement, enhancing CD8^+^ T cell-mediated antitumor immunity [147]. Notably, vaccines expressing immature laminin receptor proteins are linked to sustained protective responses mediated by CD8^+^ T cells [148]. Applying elastin-like polypeptides directly to tumors can impede the growth of specific breast cancers and reduce the incidence of lung metastasis (Fig. 5D) [149].

### Enhancing the efficacy of immunotherapy with nanocarriers targeting and degrading the ECM

In addition to the previously mentioned combination therapies, nanocarriers have shown great potential in enhancing cancer immunotherapy outcomes. For example, nanoparticles combined with MMP-2 inhibitors have been reported to improve outcomes in cancer photodynamic immunotherapy [150]. Similarly, MMP-sensitive nanomaterials, designed to degrade at a controlled rate within the TME, facilitate the release of their therapeutic contents and enhance T cell presence at the tumor site [151]. Notably, nanovaccines incorporating laminin peptides have demonstrated efficacy in boosting peptide-specific CD8^+^ T cell responses, eliciting protective immune reactions with Th1 polarization [152]. Additionally, a HA-based nanovaccine mitigates the immunosuppressive TME by promoting robust infiltration of both CD4^+^ and CD8^+^ T cells [153]. Increased ECM stiffness is correlated with elevated levels of sGAGs, such as heparan sulfate and chondroitin sulfate, which align with an intensified immune response to these GAGs [154]. Prodrug nanoparticles using chondroitin sulfate as a base target the Golgi apparatus in tumor cells. This targeting helps to reduce immunosuppression in photodynamic immunotherapy by inhibiting the secretion of immunosuppressive cytokines [155].

## Challenges and future prospects

The pivotal role of ECM stiffness in the success and prognosis of cancer immunotherapy, as well as in the precision of delivering immunotherapeutic drugs, is increasingly recognized. Modulating ECM stiffness can significantly influence the efficacy of various immunotherapy strategies, yet this integrative approach also poses challenges in transitioning from research to clinical practice.

Firstly, accurately assessing tumor stiffness is critical for evaluating patient prognosis and responsiveness to immunotherapy. Clinicians employ a range of diagnostic techniques, including manual palpation, color Doppler ultrasound, magnetic resonance elastography, shear wave elastography, and transient elastography, to gauge soft tissue stiffness. The selection of these methods often depends on the specific type of tissue being examined. An integrated approach to these techniques is vital for a comprehensive assessment of tumor stiffness. The stiffness of the ECM, predominantly determined by collagen content and cross-linking, can be indirectly measured through tools like mass spectrometry and Sirius Red staining [3]. However, further research is essential to identify the most accurate and non-invasive methods for measuring tumor stiffness, particularly for early detection.

Secondly, there is an urgent need to develop more inhibitors targeting mechanotransduction signaling pathways or ECM-degrading agents to optimize the co-delivery of immunotherapeutic drugs. Bintrafusp Alfa (M7824), which targets PD-L1 and the mechanotransducer TGF-β, is currently under clinical investigation. Although initial trials have been promising, comprehensive comparisons with standard immunotherapies are crucial [156–164]. The exploration of other immunotherapy drugs targeting the ECM is in its nascent stages, underscoring the need for extensive clinical trials to evaluate the safety and efficacy of these potential inhibitors (Table 1).

**Table 1:** Published clinical trials of immunotherapy combined with inhibitors of molecules associated with ECM stiffness.

Target type	Targets	Cancer type	Immuno-therapy type	Key molecules in immunotherapy	Therapy drugs	Efficacy	Phase	NCT number	Number of patients	Ref.
Mechano-transduction signal pathway	TGF-β	Head and neck cancer	ICB	PD-L1	M7824	PR (35.7%)	I/II	NCT04247282	14	[156]
	TGF-β	HNSCC	ICB	PD-L1	M7824	PR (12.5%), SD (12.5%)	I	NCT02517398	32	[157]
	TGF-β	Esophageal adenocarcinoma	ICB	PD-L1	M7824	PR (10%), SD (10%)	I	NCT02699515	30	[158]
	TGF-β	Esophageal adenocarcinoma	ICB	PD-L1	M7824	PR (20%), SD (13.3%)	I	NCT02517398	30	[159]
	TGF-β	Biliary tract cancer	ICB	PD-L1	M7824	CR (6.7%), PR (13.3%), SD (20%)	I	NCT02699515	30	[162]
	TGF-β	HPV-associated malignancies	ICB	PD-L1	M7824	CR (8.5%), PR (22%), SD (13.6%)	I/II	NCT02517398, NCT03427411	59	[163]
	TGF-β	Colorectal cancer	ICB	PD-L1	M7824	PR (3.1%), SD (3.1%)	I	NCT02517398	32	[164]
	TGF-β	Solid tumors	ICB	CD73	GS-1423	PR (4.8%), SD (33.3%)	I	NCT03954704	21	[165]
	TGF-β1, TGF-β2	Ovarian cancer	TCV	Autologous tumor cell vaccine	Gemogenovatucel-T	N.A.	IIb	NCT02346747	47	[166]
	TGF-β2	Non-small cell lung cancer	TCV	Allogeneic tumor cell vaccine	Belagenpumatucel-L	N.A.	III	NCT00676507	270	[167]
	TGF-βRII	Solid tumors	ICB	PD-L1	SHR-1701	CR (1.7%), PR (10.7%), SD (19.8%)	I	NCT03710265	121	[168]
	TGF-βRII	Prostate cancer	ACT	CAR-T	DnTGF-βRII-T2A-Pbbz CAR T cells	N.A.	I	NCT03089203	17	[169]
	TGF-βRII	Prostate cancer	ACT	CAR-T	CART-PSMA-TGFβRDN cells	N.A.	I	NCT03089203	14	[170]
	FAK	Pancreatic ductal adenocarcinoma	ICB	PD-L1	Defactinib, Pembrolizumab, Gemcitabine	PR (5%) SD (75%)	I	NCT02546531	20	[171]
CAF	FAP	Solid tumors	ICB	PD-L1	RO7122290, Atezolizumab	CR (1.7%) PR (7.8%)	I	N.A.	115	[172]
	FAP	Pleural mesothelioma	ACT	CAR-T	Anti-FAP-Δ-CD28/CD3ζ CAR T cells	SD (100%)	I	NCT01722149	1	[130]
	FGFR4	Hepatocellular carcinoma	ICB	PD-L1	BLU-554, CS1001	CR (25%) PR (25%) SD (50%)	Ib/II	NCT04194801	4	[173]
	FGFR1–4	Urothelial carcinoma	ICB	N.A.	Erdafitinib	ORR (59.1%)	II	NCT02365597	22	[174]

*ACT* adoptive cell therapy, *CAF* cancer-associated fibroblasts, *CR* complete response, *FAK* focal adhesion kinase, *FGFR* fibroblast growth factor receptor, *ICB* immune checkpoint blockade, *HNSCC* head and neck squamous cell carcinoma, *HPV* human papillomavirus, *N.A.* not available, *PR* partial response, pts patients, *SD* stable disease, *TCV* therapeutic cancer vaccine.

Thirdly, it is imperative to devise strategies that minimize the side effects of inhibitors targeting mechanotransduction pathways. Given the essential roles of molecules like integrins, TGF-β, and actin in maintaining tissue homeostasis, their inhibition could lead to unintended physiological consequences. Enhancing the specificity of these drugs is critical. One potential approach involves the use of nanoparticles for targeted drug delivery specifically to stiff tumor regions, thereby sparing the surrounding healthy tissues. Additionally, exploring binding sites on challenging proteins such as Rho could broaden the spectrum of combinational treatments with immunotherapy.

Fourthly, an innovative direction in cancer therapy involves targeting CAFs. Transforming CAFs into anti-tumor fibroblasts is crucial for maintaining the mechanical balance of the ECM. Contemporary strategies encompass TCVs targeting the CAF-activating protein FAP, CAR-T cell therapies, oncolytic viruses targeting CAFs, inhibiting cancer cell mechanotransduction to reduce TGF-β secretion, and focusing on pathways such as CXCL12-CXCR4, JAK-STAT3, and Hedgehog.

Lastly, a significant gap in current research is the lack of biomaterials that accurately mimic the complex composition of the ECM. Studies often rely on hydrogels derived from single ECM components, which may not faithfully represent the conditions in human tumors. While natural ECM hydrogels, often sourced from murine tumor collagen IV, provide a closer approximation to in vivo conditions, their dissimilarity to human tumor environments limits their applicability. Consequently, the development of more precise biomaterials and effective animal models is paramount for advancing the field of cancer research and therapy.

## Conclusions

In conclusion, the critical role of ECM stiffness in the realm of immunotherapy is increasingly apparent. Dysregulation of ECM stiffness profoundly influences cancer initiation and progression, underscoring its significance in oncological research and treatment. Interdisciplinary collaborations, bridging oncology, cell biology, physics, engineering, materials science, and nanotechnology, are pivotal in advancing therapeutic strategies that target ECM stiffness and mechanotransduction signaling pathways. These collaborative efforts are instrumental in transitioning such strategies from bench to bedside, potentially expanding the scope and enhancing the efficacy of immunotherapy in cancer treatment.

## Data Availability

The data generated are included within the manuscript.
